# The use of non-living animals as simulation models for cranial neurosurgical procedures: a literature review

**DOI:** 10.1186/s41016-020-00203-3

**Published:** 2020-07-07

**Authors:** Zahraa F. Al-Sharshahi, Samer S. Hoz, Mohammed A. Alrawi, Mohammed A. Sabah, Saja A. Albanaa, Luis Rafael Moscote-Salazar

**Affiliations:** 1 Department of Neurosurgery, Neurosurgery teaching Hospital, Baghdad, Iraq; 2 College of Medicine, University of Baghdad, Baghdad, Iraq; 3 RED LATINO, Latin American Trauma & Intensive Neuro-Care Organization, Bogotá, Colombia

**Keywords:** Neurosurgery, Cranial, Simulation, Non-living, Sheep, Swine, Cow

## Abstract

Simulation plays a pivotal role in neurosurgical training by allowing trainees to develop the requisite expertise to enhance patient safety. Several models have been used for simulation purposes. Non-living animal models offer a range of benefits, including affordability, availability, biological texture, and a comparable similarity to human anatomy. In this paper, we review the available literature on the use of non-living animals in neurosurgical simulation training. We aim to answer the following questions: (1) what animals have been used so far, (2) what neurosurgical approaches have been simulated, (3) what were the trainee tasks, and (4) what was the experience of the authors with these models. A search of the PubMed Medline database was performed to identify studies that examined the use of non-living animals in cranial neurosurgical simulation between 1990 and 2020. Our initial search yielded a total of 70 results. After careful screening, we included 22 articles for qualitative analysis. We compared the reports in terms of the (1) animal used, (2) type of surgery, and (3) trainee tasks.

All articles were published between 2003 and 2019. These simulations were performed on three types of animals, namely sheep, cow, and swine. All authors designed specific, task-oriented approaches and concluded that the models used were adequate for replicating the surgical approaches. Simulation on non-living animal heads has recently gained popularity in the field of neurosurgical training. Non-living animal models are an increasingly attractive option for cranial neurosurgical simulation training. These models enable the acquisition and refinement of surgical skills, with the added benefits of accessibility and cost-effectiveness. To date, 16 different microneurosurgical cranial approaches have been replicated on three non-living animal models, including sheep, cows, and swine. This review summarizes the experience reported with the use of non-living animal models as alternative laboratory tools for cranial neurosurgical training, with particular attention to the set of tasks that could be performed on them.

## Background

Simulation may be defined as an educational strategy whereby a learner can enact a live undertaking in a virtual, controlled, hazard-free environment [1]. Simulation is a well-established practice in emergency medicine and other non-medical fields, including aeronautics and the military [2]. It also plays a pivotal role in neurosurgical training by allowing trainees to develop the requisite expertise to enhance patient safety [3–5]. A wide range of biological and non-biological materials have been used in the neurosurgical simulation, including live animals, cadavers, synthetic, and computer-based models [6]. Compared to non-biological materials, the use of non-living animals provides many other advantages, including cost-effectiveness, similarity to human anatomy, real-time haptic feedback, and availability [6]. Such models can be purchased from the local market easily and entail limited preparation and maintenance.

More significant constraints on working hours and the resultant reduction in theater time combined with the increased pressure to show competence in surgical procedures have been the impetus behind the introduction of operational performance simulators [2]. It may take years for surgical residents to learn their requisite surgical skills and laboratory training models are essential for this critical development before clinical implementation [7]

In his book *Outliers*, Malcolm Gladwell argues that a minimum of 10,000 hours of practice is required for true task mastery [8]. Although this statistic is disputable, increased experience has been shown to equate with improved outcomes [8, 9].

The increased restrictions on the use of animals in scientific experimentation have promoted the search for alternative practice models. The use of non-living animal heads does not require an institutional review board approval and may raise fewer ethical concerns than the use of living animals.

In this paper, we review the available literature on the training practices surrounding cranial procedures or approaches applied to non-living animal heads for neurosurgery to understand how trainees could best develop these skills.

## Literature search

A search of the PubMed Medline database was performed to identify studies that examined the use of non-living animals in cranial neurosurgical simulation. The following algorithm was used: (non-living OR cadaveric OR Cadavers) AND (Animal OR Cattle OR Swine OR Beef OR pig OR Lamb OR Rat OR Rodent OR Rabbit) AND (Cranium OR Head OR Brain) AND (Craniotomy OR Endoscope OR Training OR Simulation) AND (Neurosurgery OR Neurosurgical procedure OR neurosurgical approaches). Two additional filters were applied for articles published between 1990 and 2020 and those published in the English language only. The inclusion criteria were (1) simulation of cranial neurosurgical approaches or procedures, (2) non-living animals used as models, and (3) trainee tasks described in detail. The exclusion criteria were (1) simulations of spine procedures/approaches, (2) studies experimenting the use of an instrument, (3) anatomical studies, and (4) studies using a simulator other than non-living animals. Our initial search yielded a total of 70 results. All the abstracts were for inclusion by two independent reviewers. The references section of each article was screened for additional studies. Ultimately, only 22 articles were included for qualitative analysis. Data was collected from each study in view of the following questions: (1) what animals have been used? (2) what types of neurosurgical approaches have been simulated? (3) what were the trainee tasks? (4) and what was the experience of the authors with these models?

## Non-living animals used in 16 different cranial approaches

The first article was published in 2003. A total of 16 different neurosurgical procedures were simulated on non-living animal heads, as follows: (1) bifrontal and fronto-lateral approaches, (2) the approach to the circle of Willis, (3) the interhemispheric-transcallosal approach to the lateral ventricle, (4) the approach to the orbit and optic nerve, (5) the middle cranial fossa dissection, (6) the midline posterior fossa approach, (7) the exploration of the cranial nerves in posterior cranial fossa, (8) the retrosigmoid approach, (9) endoscopic surgery of the cerebellopontine angle, (10) microsurgical and endoscopic skull base surgery, (11) brain tumor resection, (12) surgery for craniosynostosis, (13) surgery for a cerebral aneurysm, (14) intraoperative ultrasound, (15) Sylvian fissure microdissection, and (16) microsurgical sulcal-cisternal and fissural dissection. These simulations were performed on three types of animals, namely sheep, cow, and swine. The use of sheep cranium as a simulator was reported in 8 studies, the use of cows and swine was reported in the remaining 14 studies with equal frequency. All authors designed specific, task-oriented approaches and concluded that the models used were adequate for replicating the surgical approaches and improving the trainees’ skills.

## Task-oriented simulation training

The importance of simulation is well-ingrained in the field of surgical training [3, 5]. Various models, both synthetic and biologic, are available as simulators [6]. Thus far, 16 cranial neurosurgical approaches have been simulated on non-living animal models (Table 1). Sheep, cow, and swine have been the three types of animals used thus far, with encouraging feedback. Compared to humans, animal training models have anatomical differences in terms of size, topographic anatomy, neuronal, and vascular relations. However, the models are useful in helping trainees to acquire the manual dexterity and to perform the surgical steps involved in a variety of cranial approaches. Many of these differences are also negligible in the context of microscopic surgery [1, 2, 4, 12].

**Table 1:** The types of neurosurgical approaches/procedures that were simulated on non-animal head models

No.	Date	Author(s)	Animal’s head used	Procedure applied
1	2003	Borucki and Szyfter [10]	Swine	Endoscopic surgery of the cerebellopontine angle
2	2005	Hicdonmez et al. [11]	Cow	Interhemispheric-transcallosal approach to the lateral ventricle
3	2006	Hicdonmez et al. [12]	Cow	Approach to the circle of Willis
4	2006	Hicdonmez et al. [13]	Sheep	Posterior fossa approach
5	2006	Hicdonmez et al. [14]	Sheep	Surgery for craniosynostosis
6	2008	Hamamcioglu et al. [15]	Sheep	Microneurosurgical dissection of cranial nerves in posterior fossa
7	2010	Regelsberger et al. [16]	Swine	Artificial tumor created by injection of colored fibrin glue
8	2011	Olabe et al. [17]	Swine	Cerebral aneurysm creation, clipping and vessel reconstruction
9	2012	Yatomi et al. [18]	Swine	Terminal aneurysms creation
10	2013	Turan Suslu et al. [19]	Cow	Retrosigmoid approach
11	2013	Scholz and Vavruska [20]	Sheep	Intraoperative ultrasound in neurosurgery
12	2014	Aurich et al. [21]	Swine	Transcallosal approach to the lateral ventricle, middle cranial fossa dissection, posterior cranial fossa dissection
13	2014	Silva et al. [22]	Swine	Skull base approaches
14	2014	Altunrende et al. [23]	Sheep	Approach to the orbit and the optic nerve
15	2014	Mücke et al. [24]	Sheep	Approach to the orbit and the optic nerve
16	2014	Vavruska et al. [25]	Sheep	Intraoperative ultrasound in neurosurgery
17	2015	Tatarli et al [26]	Cow	Bifrontal and fronto-lateral approaches
18	2015	Kamp et al. [27]	Sheep	Brain tumor resection
19	2018	Gökyar [28]	Cow	Sylvian fissure microdissection
20	2019	Elsayed et al. [29]	Swine	Posterior fossa surgery
21	2019	Altun et al. [30]	Cow	Microsurgical intrinsic and extrinsic brain tumor surgery
22	2019	Gökyar et al. [31]	Cow	Microsurgical sulcal-cisternal and fissural dissection

Tatarli et al. [26] simulated two of the most common neurosurgical approaches, the bifrontal and fronto-lateral, using a non-living cow as a model. The authors described a three-step approach, including craniotomy, dural opening, and eventually dissection and identification of the following structures: olfactory nerves, olfactory cisterns, internal carotid artery, optic nerves, optic chiasm, and the optic canal. The authors concluded that the dissection process familiarized trainees with the microsurgical techniques required for the bifrontal and fronto-lateral approaches. Also, the target vascular and other structures were readily identifiable.

Hicdonmez et al. [12] planned a 4-step procedure to replicate the approach to the circle of Willis on a fresh non-living cow brain. They dissected the internal carotid artery and its proximal branches, the optic nerve, the optic chiasm, and the pituitary stalk [5] The authors concluded that the corpse cow brain represented a high-fidelity model that allowed the intracranial vessels and nerves to be precisely dissected simulating intracranial microneurosurgical procedures performed on the human brain.

The interhemispheric-transcallosal approach to the lateral ventricle was simulated by Hicdonmez et al. [11] and Aurich et al. [21], who used cadaveric cow and swine heads, respectively. The simulation included the following structures: the interhemispheric fissure, callosomarginal arteries, cingulate gyri, corpus callosum, pericallosal arteries, lateral ventricle, choroid plexus, septal and thalamostriate veins, and the foramen of Monro. The authors reported that both models perfectly simulated the standard micro neurosurgical steps of the interhemispheric-transcallosal approach to the lateral ventricle.

Altunrende et al. [23] and Mücke et al. [24] simulated an approach to the orbit and the optic nerve of the non-living sheep cranium. The simulation consisted of two parts; one to access the intraorbital structures, a two-step upper orbitotomy approach, and another to dissect the intracranial part of the optic nerve, a three-step, standard frontal craniotomy approach. During the simulation process, the trainees could identify the following structures: supraorbital nerve, periorbita, Sylvian fissure, carotid and basal cisterns, optic nerve, and the optic canal. The authors concluded that simulation models provided valuable assets for both neurosurgery and ophthalmology trainees.

Middle cranial fossa dissection was simulated by Aurich et al. [21] on cadaveric swine head. The middle meningeal artery, and the V2 and V3 branches of the trigeminal nerve were reached through extradural dissection of the middle fossa. Then, the lateral sulcus was dissected to visualize the optic nerve and internal carotid artery. The authors found that the pig model served as an adequate simulation model and had great value for the acquisition and refinement of surgical skills.

The midline posterior fossa approach was simulated by Hicdonmez et al. on sheep [15] and by Aurich et al. on swine [21]. The simulated approach involved the cisterna magna, fourth ventricle, Sylvian aqueduct, brainstem, and lower cranial nerves. Both sheep and swine were found to be useful simulators for necessary steps in standard posterior fossa surgery, especially in infants and children.

Hamamcioglu et al. [15] described a four-step approach to the simulation of the micro neurosurgical dissection of cranial nerves in the posterior fossa on a fresh cadaveric sheep brain. The authors concluded that the model was successful.

The retrosigmoid approach was simulated on the silicone-injected cow brain by Turan Suslu et al. [19] and on swine by Aurich et al. [21]. The cerebellum was partially resected to expose the cerebellopontine angle. Drilling of the internal auditory canal could also be accomplished. Both models were found to be useful educational tools. Silva et al. [22] and Borucki and Szyfter [10] found cadaveric swine heads to be useful simulators for microsurgical and endoscopic skull base surgery.

Brain tumor resection was simulated on cadaveric animals using different agents. Such agents may be localized to form sharply delimited masses that mimic cerebral metastases or be diffuse to migrate to the surrounding brain tissue and resemble glioma. These artificial lesions can then be excised using the standard brain tumor microsurgical steps [16, 27, 30].

More recently, Gökyar et al. [31] used fresh cadaveric cow brains to microscopically dissect the Sylvian cisterns, interhemispheric fissure, and hemispheric sulcus. The authors concluded that the models were useful aids for microsurgical training.

Elsayed et al. [29] used pig heads to expose the course of the facial and vestibulocochlear nerves as they emerged from the brain stem in the cerebellopontine angle, then within the internal carotid artery. Gökyar [28] used a fresh cadaveric cow brain to perform Sylvian fissure microdissection while preserving neighboring neurovascular structures. In both studies, the authors agreed on the viability of the models in their chosen approaches.

More advanced simulations were also applied to cadaveric animal heads, including craniosynostosis [14] surgery, cerebral aneurysm surgery [17, 18], and intraoperative ultrasound training [20, 25], all of which had a beneficial effect on the development of trainee skills.

Our literature review has demonstrated the utility of various non-living animal specimens as simulation models for cranial microsurgical procedures. We analyzed these studies with particular attention to the set of tasks that could be practiced on each of them. The use of sheep cranium as a simulator was reported in 8 studies, with the remaining 14 studies reporting the use of cow and swine with equal frequency. However, in all individual studies, none of the experiments compared the use of these animal simulators from a logistical or neurosurgical stimulation point of view. To allow an informed, task-specific model choice, a comparative study that evaluates the utility of these three different specimens while controlling for the type of surgical procedure, approach, and skill set is required.

## Conclusion

Non-living animal models are an increasingly attractive option for cranial neurosurgical simulation training. These models enable the acquisition and refinement of surgical skills, with the added benefits of accessibility and cost-effectiveness. To date, 16 different microneurosurgical cranial approaches have been replicated on three non-living animal models, including sheep, cows, and swine. This review summarizes the experience reported with the use of non-living animal models as alternative laboratory tools for cranial neurosurgical training, with particular attention to the set of tasks that could be performed on them.

## Data Availability

Not applicable
